# Adaptive Activity and Environment Recognition for Mobile Phones

**DOI:** 10.3390/s141120753

**Published:** 2014-11-03

**Authors:** Jussi Parviainen, Jayaprasad Bojja, Jussi Collin, Jussi Leppänen, Antti Eronen

**Affiliations:** 1 Department of Pervasive Computing, Tampere University of Technology, FI-33101 Tampere, Finland; E-Mails: jayaprasad.bojja@tut.fi (J.B.); jussi.collin@tut.fi (J.C.); 2 Nokia Technologies, FI-33721 Tampere, Finland; E-Mails: jussi.ar.leppanen@nokia.com (J.L.); antti.eronen@nokia.com (A.E.)

**Keywords:** mobile sensing, classifier design and evaluation, activity recognition, environment recognition, Bayes classifier, adaptation, pervasive computing

## Abstract

In this paper, an adaptive activity and environment recognition algorithm running on a mobile phone is presented. The algorithm makes inferences based on sensor and radio receiver data provided by the phone. A wide set of features that can be extracted from these data sources were investigated, and a Bayesian maximum *a posteriori* classifier was used for classifying between several user activities and environments. The accuracy of the method was evaluated on a dataset collected in a real-life trial. In addition, comparison to other state-of-the-art classifiers, namely support vector machines and decision trees, was performed. To make the system adaptive for individual user characteristics, an adaptation algorithm for context model parameters was designed. Moreover, a confidence measure for the classification correctness was designed. The proposed adaptation algorithm and confidence measure were evaluated on a second dataset obtained from another real-life trial, where the users were requested to provide binary feedback on the classification correctness. The results show that the proposed adaptation algorithm is effective at improving the classification accuracy.

## 1. Introduction

The design of novel applications for modern smartphones has boomed in the past few years. This is partly due to easy access to information sources previously unavailable, such as the Global Positioning System (GPS) and motion sensors. For application designers, the smartphone environment is very different from the earlier personal computer world. Applications are running continuously, and the environment and activity of the user changes many times a day. Context information is often required to provide only the most relevant information or services to the user. In the present paradigm, the application requires the user to provide this information, such as ‘I am in a meeting’. Undoubtedly, it would be more convenient if this context information could be inferred automatically. For this reason, context recognition using the built-in sensors of modern mobile phones is an active research topic. The research problem is far from trivial, as the sensors provide only indirect information; there is no sensor for directly detecting ‘meetings’ or ‘jogging’. Furthermore, the required accuracy for the recognition is relatively high, and misclassifications can lead to very annoying user experiences. In this paper, we introduce a framework for automatic activity and environment classification that can be easily implemented and evaluated on any modern smartphone.

In this paper, we consider activity and environment recognition from mobile phone sensor and radio receiver data. The goal of activity recognition algorithms is to output information on the activity of the user. In particular, we consider algorithms that try to classify the physical activity of a user, such as walking, running, driving a car, riding a bicycle or being still. By environment recognition, we mean the automatic recognition of user surroundings, such as whether the user is in a meeting, at the office or inside a vehicle.

Existing literature on this topic includes many interesting examples of what can be done with sensor systems carried by the user. Altun *et al.* present a study of classifying human activities using body-worn inertial and magnetic sensors [1]. The authors perform a comparative study of different methods. The results in the paper indicate that, in general, Bayesian decision making results in the highest correct classification rate with a relatively small computational cost. Gu *et al.* describe a body sensor network for activity recognition for multiple users [2]. Könönen *et al.* describe several classification and automatic feature selection algorithms, which are compared in the problem of context recognition [3]. Reddy *et al.* present a classification system that uses a mobile phone with a built-in GPS receiver and an accelerometer [4]. The system recognizes the following transportation modes: whether an individual is stationary, walking, running, biking or in motorized transport. In the UPCASEproject [5,6], multiple sensors, such as accelerometers, light, sound, humidity, temperature and GPS sensors, were connected to a mobile phone via Bluetooth. Decision trees were used to obtain the activity (walking, running, standing or lying) of the user. The system also detects whether the device is indoors or outdoors by using information on the availability of GPS signals. After recognition, the context of the user can be published on social networks, such as Twitter or Facebook. The project CenceMe [7] also describes an activity recognizer where the results can be sent to social networks. The works in [8,9] present activity-based pattern matching in different mobile environments. Bancroft *et al.* use a foot-mounted inertial measurement unit (IMU) with a GPS to detect activity and the environment of the user [10]. In [11], Pei *et al.* use location and motion tracking to build a context-aware system. Susi *et al.* present in [12] motion mode recognition for pedestrian dead reckoning. A smartphone-based lightweight hierarchical activity recognition framework is presented in Han *et al.* [13], where the recognition of 15 activities is done using the accelerometer, gyroscope, proximity sensor and GPS modules.

Our novel contribution to prior work is that we introduce an algorithm for user-specific adaptation of the context model parameters from user-friendly binary feedback. Furthermore, the introduced adaptation method is able to provide a confidence measure about the correctness of a classification for the host application.

This paper is organized as follows. In Section 2, we present the data used for training and testing the proposed classifier algorithms. Section 3 describes the features that were extracted from the collected data. Section 4 presents the feature compression algorithm used. An introduction to our classifier is presented in Section 5. In Section 6, we introduce our adaptation method. Section 7 presents the results from multiple classifiers and the results obtained with the proposed adaptation method. Finally, we conclude the paper in Section 8.

## 2. Data Collection

To be able to train and evaluate the classification system, a comprehensive annotated data set was collected. We chose Nokia N900 mobile phones for data collection, the main reason being easy access to sensor data via application programming interfaces and the open source nature of the Maemo platform. Two data collection campaigns were arranged. The first campaign, with 21 users, was arranged to collect a basic training set with full annotations, providing enough data for the design and validation of the feature extraction and pattern recognition algorithms. During the first campaign, the users were requested to manually input their current activity and environment from the mobile phone user interface (UI). The second campaign, with 10 users, was arranged for testing the adaptation of algorithm parameters for each individual user. The campaign was done with context recognition software running and with only binary yes, no input, indicating whether or not a recognition result was correct or not, required from the user. It should be noted that the first campaign required much more effort from the test persons since the users were providing full annotation compared to the binary yes/no answers in the second campaign.

### 2.1. The First Data Collection Campaign

**Figure 1.**
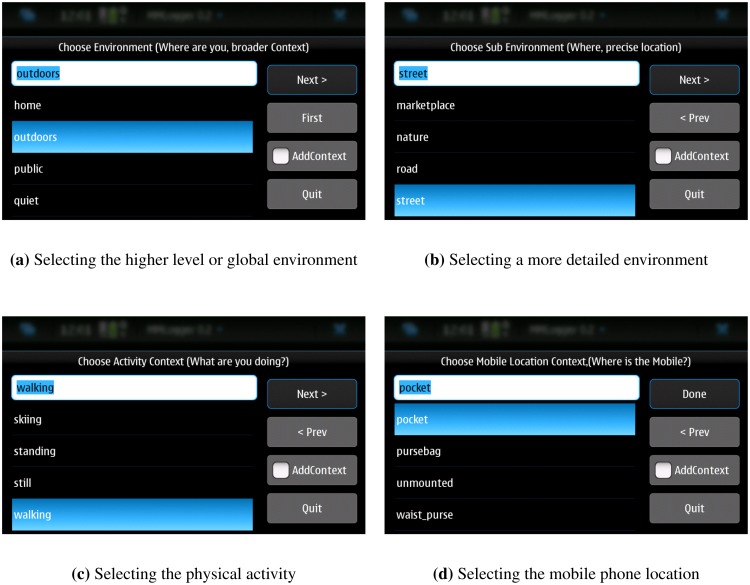
Data collection program.

In the first data collection campaign, the users were requested to carry the phone along with them as they normally would during three to four weeks. Our software would then ask the users to annotate their environment (e.g., outdoors, street), activity (e.g., walking) and the location of the phone (e.g., pocket) at set intervals. Once the annotation was input the phone sensors would then record data for two minutes. The default time interval between recordings was 20 min, but could be set by the user to be from a minimum of 10 min to a maximum of 60 min. The annotations were tagged to the recorded sensor data by a file name convention. The user interface used by the users to provide the annotations is depicted in Figure 1. There was a fixed set of predefined environments, activities, and phone locations available but the users could also add their own if they thought none of them matched their own situation.

After the user provided an annotation for the context, the sensors on the device were activated and a short period of data collection was performed. The used sensors and statistics of the obtained data are presented in Tables 1 and 2. The collected sensor data is described in more detail below.

- *Accelerometer*: The sampling rate of the 3-axis accelerometer was approximately 100 Hz. Data was collected for one minute.
- *GPS*: Three minutes of GPS data was collected with a 1 Hz sampling rate. The recorded data included position, velocity, satellite elevation and azimuth angles and signal to noise ratio.
- *WLAN*: WLAN signal strengths and WLAN station identifiers (IDs) and Media Access Control (MAC) addresses were recorded. Samples were collected for one minute and the sampling interval was approximately 12 s. Thus, five scans were performed during one minute.
- *Bluetooth*: A single scan of the Bluetooth environment was performed, and the names and addresses of the visible Bluetooth devices were logged.
- *GSM/3G Cell information*: Global System for Mobile Communications (GSM) or 3rd generation mobile communications (3G) location area codes (LAC) and cell IDs were recorded
- *Audio*: One minute of audio with a single mono channel with 16-bit resolution at 16 kHz sampling rate was recorded.

### 2.2. Data Campaign for Adaptation

The second campaign was done using Nokia N900 devices wherein the developed classification algorithm for activity (classes: *running, bicycling, walking, standing, table, vehicles*) and environment (classes: *office, nature, street/road, home, restaurant/pub/cafe*) was running. Algorithms are described in detail in the Section 5.

**Figure 2.**
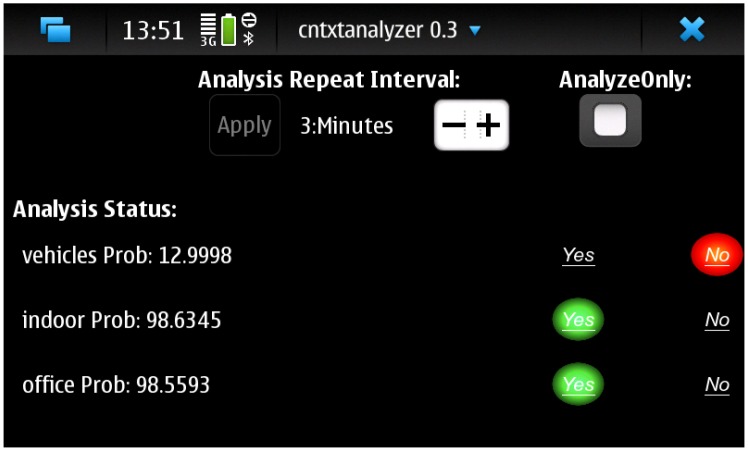
During the second trial, user were able to give input on whether the classification was correct or incorrect.

The user feedback was requested by displaying a green button for “yes” and a red button for “no” right after the classification result was available. The user interface of the program is depicted in Figure 2. If the user did not provide an answer, the data was not used for adaptation. For this data collection campaign, ten users who did not participate the first campaign were selected for two week trial. In total, there were 2674 classification results with the version that asks the feedback.

## 3. Feature Extraction

This section presents the features extracted from the mobile phone sensor and radio receiver data. Multiple features were implemented and used, as the redundancy can be removed using a compression algorithm presented in Section 4.

### 3.1. GPS Features

The following features are extracted from the GPS receiver.

- The median carrier to noise ratio value of one minute of GPS data. The carrier to noise ratio is an estimate of the received power most GPS/GNSS (Global Navigation Satellite System) receivers provide for each tracked satellite. A low carrier to noise ratio indicates that there are obstacles such as building walls in the satellite-receiver path. The carrier to noise values are much lower indoors than outdoors [14].
- The minimum elevation angle value from the satellites that are used for getting the GPS position fixes. The elevation angle is the angle between the local horizontal plane and the user-satellite vector. In urban canyons satellites with a small elevation angle are rarely tracked.
- The maximum speed value from one minute of GPS data. Preferably a Doppler-based speed estimate computed by the receiver, but can also be derived from two consecutive position fixes. High speed values indicate that the device is in a moving vehicle.
- The best horizontal accuracy value of the GPS position fixes, *i.e.*, the smallest horizontal accuracy value that the GPS device outputs. Most receivers provide this kind of value, the best accuracy is obtained in rural areas with a clear sky view.
- Time to first fix (TTFF), *i.e.*, the time passed from the time of power-up to the instant when the first location measurement is obtained.

### 3.2. WLAN Features

The features extracted from the WLAN receiver are

- The number of unique MAC addresses.
- The number of unique station names. Usually in public areas one observes more MAC addresses than station names due to having several access points connected to the same WLAN network, whereas in private places such as homes one usually observes less MAC addresses per station name.
- The average signal strength on a scale from one to five.
- The average signal strength given on a decibel scale.
- The standard deviation of the signal strength on a scale from 1 to 5. A high standard deviation usually indicates that user is on the move.
- The standard deviation of the signal strength on a decibel scale.
- The maximum signal strength on a decibel scale. A high signal strength usually indicates that the user is indoors.
- The maximum signal strength on a scale from 1 to 5.

### 3.3. Bluetooth Features

A single feature related to the Bluetooth radio environment is used. The feature is the number of visible Bluetooth devices. A high number of Bluetooth devices indicates that the user is in a public place.

### 3.4. Cellular Network Environment Related Features

The cellular base stations to which the mobile phone connects during a minute are logged. From the logged data, the following statistics are used as features:

- The number of unique Cell IDs (base transceiver station identifiers).
- The number of unique location area codes (LAC).
- The number of Cell ID changes per minute.
- The number of LAC changes per minute.
- The standard deviation of the strength of the signal to the transceiver station the mobile phone is connected.

### 3.5. Accelerometer Features

To remove the effect of orientation of the mobile phone, the Euclidean norm of the three dimensional accelerometer signal is used. The following features are extracted from the accelerometer signal norm:

- The variance of acceleration.
- The value of the maximum spectrum magnitude peak.
- The bin index of the spectrum magnitude maximum value.
- The difference between the maximum and minimum spectrum magnitude value.
- The number of zero crossings.

### 3.6. Audio Features

For the audio features, likelihoods given by a global model trained for the audio data are used. The likelihood features are obtained as follows. First, during an offline training phase, 13 mel-frequency cepstral coefficients (MFCCs) along with their first- and second-order derivatives are calculated from audio training data [15]. The MFCCs are calculated from audio data recorded at 16 kHz sampling rate using a window of 30 ms and a frame-skip of 40 ms. After this, a single Gaussian mixture model (GMM) with 32 component densities is trained. Once the above offline training phase is complete, the likelihood features can be calculated for input audio data. This is done by, first, calculating MFCCs for the input audio data and then calculating the mixture likelihoods of the GMM for the MFCC feature vectors. The mixture likelihoods for the whole audio clip are then output as the likelihood features. Thus, for each input audio clip, we obtain a vector of 32 likelihood values. By using likelihood features we can represent a whole audio clip with a single feature vector instead of a sequence of MFCC vectors. An alternative way to represent an audio clip with a single feature vector would be to, for example, use the mean of the calculated MFCCs. Also, a covariance matrix could be calculated. However, information is lost when using only the mean of the MFCCs and for shorter audio clips, there might not be enough data to reliably calculate the variances or a covariance matrix.

## 4. Feature Compression

Given that a smartphone includes a multitude of relevant sensors for the classification task, the number of features is inevitably very high. To avoid numerical problems and make the adaptation more straightforward it is necessary to ignore redundant and irrelevant features. In order to reduce the dimensionality of the feature space, we use a method based on a transformation with two stages [16,17].

First, all the training features are concatenated in a *k* − *by* − *n* matrix *G*, where *k* is the number of features and *n* the number of feature vectors. In the following, by covariance matrix Σ we mean the sample covariance matrix obtained from the training data *G.*

The averaged within-class covariance matrix is defined as

(1) $$S_{W}=\frac{1}{C}\sum_{j=1}^{C}\sum_{j}$$

where *C* is the number of classes (in this work we have multiple sets of classes *C*, e.g., the activities and environments have separate training data sets and classifiers. Nevertheless, for notational simplicity here we only use a single set of classes *C*). and Σ*_j_* is the covariance matrix of class *j.* The required transformation matrix *R*_1_ is obtained using the eigenvectors (in the matrix *U*) and the rank of *S_w_* (*r*):

(2) $$R_{1}=\Lambda_{r}^{-\frac{1}{2}}U_{r}^{T}$$

where the diagonal matrix Λ contains the *r* eigenvalues of *S_w_*, and thus *R*_1_ *S_w_* $R_{1}^{T}=I$ ,an *r*−*by*−*r* identity matrix. This transformation scales the raw features conveniently. The dimension reduction removes redundant features, for example those that are constant all the time.

The next phase is to compress the class mean information. The between-class covariance matrix is defined as

(3) $$S_{B}=\frac{1}{C}\sum_{j=1}^{C}(\mu_{j}-\mu ){\left(\mu_{j}-\mu \right)}^{T}$$

where ***μ*** is the mean of the within-class means ***μ****_j_*. *S_B_* describes how far apart the class means are from each other. In the second phase, we seek for a transformation $R_{2}=V_{v}^{T}$ that diagonalizes *S_B_*:

(4) $$V_{v}^{T}(R_{1}S_{B}R_{1}^{T})V_{v}={\tilde{\Lambda }}_{v}$$

where *ν* is the rank of *R*_1_*S_B_* $R_{1}^{T}$ and the matrix *V_v_* = [**v**_1_,…, **v***_v_*] consists of *ν* eigenvectors of *R*_1_*S_B_* $R_{1}^{T}$ (*i.e.*, eigenvectors of *S_B_after* the first transformation *R*_1_). Similarly, Λ̃*_v_* is a diagonal matrix containing *ν* eigenvalues.

There are at most *C* − 1 non-zero eigenvalues (after the transformation the number of features is reduced at maximum to *C* − 1) and after the transformation *R*_2_*R*_1_ the first feature vector can be considered as the most important for the classification purposes. This geometric interpretation of linear discriminant analysis (LDA) is very useful in displaying the data, and it is straightforward to implement. The matrix *R* = *R*_2_*R*_1_ can be computed off-line, and the software in the mobile phone only needs to perform a matrix-vector multiplication for the raw features. In addition, some features clearly do not follow a Gaussian distribution (number of WLAN APs, for example), but after the feature compression we can quite safely work with Gaussian models.

## 5. Classification

For the classification task, we use a Bayesian maximum a posteriori classifier (MAP). For comparison, the classification results are also calculated using support vector machines (SVM) and decision trees (DT). Nevertheless, the probabilistic Bayesian MAP method has the following advantages:

(1) If the features follow Gaussian distribution the classifier is straightforward to implement. Furthermore, the output is optimal in a probabilistic sense, and it is possible to derive a proper confidence measure for the classification result.
(2) Adaptation can be performed by changing the parameters of the class distribution.
(3) The Bayesian approach allows a straightforward implementation of recursive filters. For example, data from multiple users can be combined recursively from the same geolocation.

With the underlying Gaussian assumption, the pattern recognition problem simplifies to a discrimination between *p* multivariate normal populations. First, a training data set with known states is collected to obtain

(5) $$z_{j}∼N(\mu_{j},\Sigma_{j})$$

the distribution of the observed *q* − *by* − 1-vector **z** given that the observation comes from the class *j.* At this stage, the mean vector ***μ****_j_*(*q* × 1) and the covariance matrix Σ*_j_*(*q* × *q*) are here assumed to be perfectly known (very representative training data set). We also assume that for all classes Σ*_j_* > 0. If Equation (5) holds, the density function of **z***_j_* is

(6) $$f_{z_{j}}(z;\mu_{j},\Sigma_{j})=\frac{1}{{\left(2\pi \right)}^{q/2}\sqrt{\left|\Sigma_{j}\right|}}\exp[-\frac{{\left(z-\mu_{j}\right)}^{T}\Sigma_{j}^{-1}\left(z-\mu_{j}\right)}{2}]$$

A new observed **z = z***_x_* can then be classified by maximizing Equation (6) over all the classes *j* = 1…*p.* To assign a probability to the classification result, we need to use unconditional prior probabilities *P*(*C* = *j*), and assume that all the possible classes are included. Then the probabilities for all the classes are obtained from the Bayes' rule:

(7) $$P(C=j|z_{x})=\frac{f_{z_{j}}(z_{x})P(C=j)}{f_{z}(z_{x})}$$

where *f***_z_** is the unconditional density function for the observation **z**. However, Equation (7) may not work well in practical classification problems as the Gaussian assumption does not necessarily hold. In addition, the class parameters ***μ****_j_*, Σ*_j_* are only estimates obtained based on incomplete training data and the list of classes is not complete – we do not have data from all the possible activities or environments. For these reasons it is necessary to modify this basic model.

### 5.1. Combining Independent Classifiers

The information provided by all the *p* likelihoods *f*(**z***_x_*; ***μ****_j_*, Σ*_j_*) of the classes *j* = 1…*p* becomes interesting when combining different classifiers, such as an audio-based classifier and radio receiver based classifiers. The motivation for this approach is that often there are specialized classifiers which operate on certain type of sensor data (e.g., audio or accelerometer). Different sensors have very different data rates and feature statistics, and often it is impossible to concatenate raw features to the same feature vectors to perform feature level fusion. Instead, if we have a classifier, the output of this classifier can be fed to another classifier as a feature vector. This makes it possible to roughly match the data rates of different sensors, as in the case of a radio receiver producing a set of data every one minute combined with the output of the audio classifier at the same rate. In addition, in this case the application software does not have access to the classifiers, but only obtains the result containing the likelihoods for all the possible classes, and the decision itself.

Thus it is convenient to use these likelihoods as features for another classifier that is running on a higher level in the software. By doing this we would not need to know any details of the original classifier. First we need to investigate how the *f*_Z_*_j_* (**z**) itself is distributed, considering *f* as a deterministic function of a random variable **z**. First, assume that the new observation **z** = **z***_x_* comes from the population class *j* = 1. To simplify the computation we take the natural logarithm of the likelihood, yielding a constant term (for each class) ln $\frac{1}{\sqrt{\left|2\pi \Sigma_{1}\right|}}$ plus the term

(8) $$-\frac{{\left(z-\mu_{1}\right)}^{T}\Sigma_{1}^{-1}(z-\mu_{1})}{2}$$

As the actual class is correct (*i.e.*, **z** and ***μ***_l_ belong to the same class), by Theorem A.85 in [18], the numerator in Equation (8) is chi-square distributed with *q* degrees of freedom.

Then, we need to study the distribution of incorrect class (*i.e.*, classifier parameters are wrong). The distributions of likelihoods for the incorrect classes is not as straightforward. We will have a quadratic form (**z** + **a**)*^T^C*(**z** + **a**), where the vector a and the matrix *C* have nothing to do with the parameters of the distribution of **z**. From [19] we find that

(9) $$P\{{\left(z+a\right)}^{T}C\left(z+a\right)\leq t\}=\sum_{k=0}^{\infty }c_{k}F(\cdot ,t)$$

where F is a chi-square cumulative distribution function (CDF). This formula can be used to obtain a numerical approximation of the CDF for the likelihoods in the case of incorrect class. The normally distributed features produce chi-squared likelihoods when the class model is correct. We also know the shape of the distribution (Equation (9)) in the case of an incorrect class. Thus, if we leave the assumption of normally distributed features aside, we can train a new classifier using directly the likelihoods of the classifier running on a lower level. Feature compression is clearly necessary when feature vectors with a non-normal degenerate distribution are added to the classifier. We used this approach to combine an existing audio-based environment classifier with the radio-based classifier developed in this study. In our existing audio classifier we could only touch the outputs of the classifier (*i.e.*, in this case likelihoods). In addition, these likelihoods were not from the same classes that were are interested in the final classification. Thus, classical ensemble methods [20] were not valid in this case.

## 6. Adaptation

It is clear that one single global model for activity or environment classification will not perform well in the case of mobile phones. There are individual differences that affect the activity monitoring between users (walking pace, step impact, bicycling speed, *etc.*). The same applies for the environment classification, for example, offices tend to have different WLAN coverage and there are differences in the audio and radio environment between cities. For that reason, adaptation via user feedback is needed. To reduce the amount of work the user has to do it is preferable to ask only binary information from the user: given the global distribution model the software outputs the classification result and prompts the user to provide a *yes* or *no* answer depending on whether the classification result was correct or not. This user input is then used to adapt the distribution parameters. A similar idea is also proposed in [3], but we will add the likelihood distribution models for the adaptation process.

### 6.1. Adaptation Algorithm

Using likelihood distributions we can derive a new optimization criteria: the *yes* answer likelihoods should be chi-square distributed with *q* degrees of freedom *after* the adaptation (this was the definition of numerator in Equation (8)). In addition, to reduce the amount of incorrect classification results, the *no* answer likelihoods should be far away (towards +∞) from this distribution.

In this paper we use the following optimization criterion: modify the distribution parameters so that the function

(10) $$f=\frac{\left|A\right|}{N(yes)}+\frac{\left|B\right|}{N(no)}$$

is minimized. It should be noted that it is possible to use other suitable adaptation functions, for example, the one proposed in [3]. Function in the paper was chosen somewhat heuristically. In our function symbols *N*(*yes*) and *N*(*no*) denote the total numbers of *yes* and *no* answers and |*A*| and |*B*| denote the number of items in the set *A* and *B*, respectively. The sets are defined as

(11) $$A=\{L_{i}(yes)|L_{i}(yes)>\chi_{95}\}$$

and

(12) $$B=\{L_{i}(no)|L_{i}(no)<\chi_{95}\}$$

Here *i* is the index of the current class and *L_i_*(*yes*) and *L_i_*(*no*) are the set of likelihood values corresponding to observations with a *yes*-label and a *no*-label, respectively, and *χ*_95_ is the point where the cumulative Chi-square distribution and has a value of 0.95. The likelihood values are

(13) $$L_{i}={\left(z-\mu_{i}\right)}^{T}s_{i}\Sigma_{i}^{-1}\left(z-\mu_{i}\right)$$

The parameters to be adapted are the scales *s_i_* and the class means ***μ****_i_*. After the adaptation, the new covariance will be *s_i_*Σ*_i_*.

The motivation for the function Equation (10) is that it resembles the Neyman-Pearson *type false positive, false negative* terminology [21,22]. Minimization of the function *f* can be thought of as minimizing the false positive and false negative errors. However, the actual classification method is not taken into account here.

In essence, the adaptation algorithm attempts to minimize the number of samples with a *yes*-label that do not fit the model distribution (in Equation (6)), meaning that they fall outside the 95% threshold. Furthermore, the algorithm attempts to minimize the number of samples with *no*-label that fit the model distribution well, meaning that they fall inside the 95% threshold. To minimize the function *f* in Equation (10), *i.e.*, to find

(14) $$\overset{\begin{array}{cc} \arg\min & f \end{array}}{s_{i}\in {\mathbb{R}}^{+},\mu \in {\mathbb{R}}^{N}}$$

We used the Matlab function *fminsearch*, which finds the minimum of the function using the simplex search method [23]. Functions *f* are implemented separately for activity and environment. The parameters to be adapted are the scale *s*, so that the new covariance will be *s*Σ, and the class means ***μ***. For *fminsearch* the scale is represented as *d* = *s*−1, so that the adaptation starts from the zero-vector. The result of *fminsearch* then tells how the parameters need to be adapted. If the adaptation is successful, we should obtain better classification results in the future. Furthermore, the likelihood distribution should approximately follow the model we have imposed - regardless of the original assumption of a Gaussian distribution.

The information contained in the sample distribution (obtained using the Matlab function *ksdensity*, for example) of yes-likelihoods and no-likelihoods is very useful for classifier diagnostics. For example, if the real feature distribution is actually multimodal, the yes-likelihood distribution should not look like a Chi-squared distribution.

### 6.2. Simulated Example

**Figure 3.**
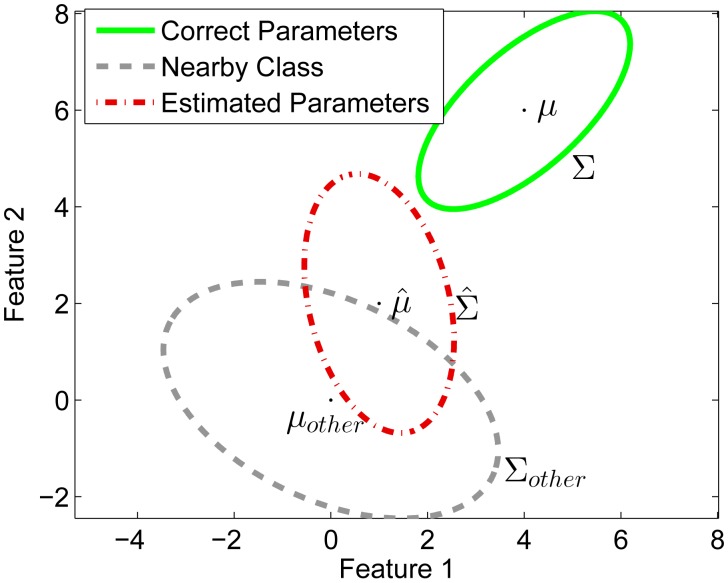
Simulated distributions.

**Figure 4.**
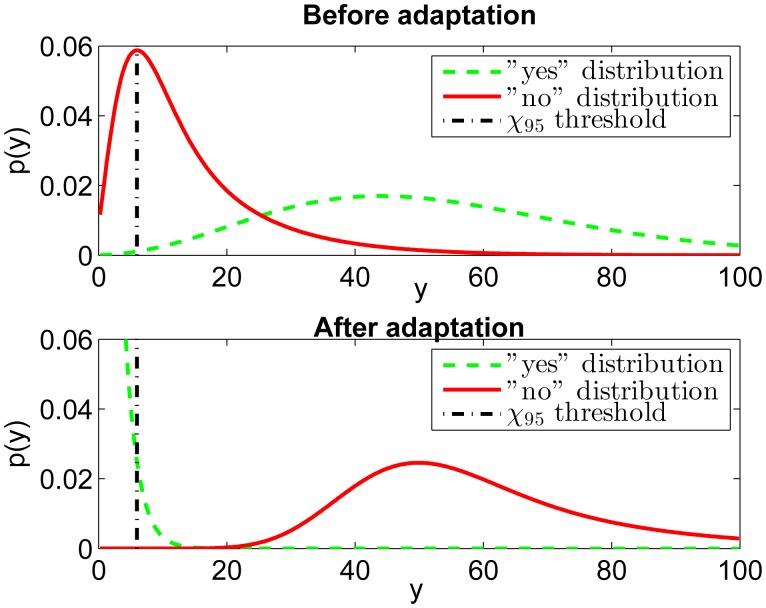
Simulated likelihood distributions before and after perfect adaptation.

To illustrate our adaptation algorithm, we present a simulated example in this section. In this simulation we assume that we have trained a MAP classifier having parameters *μ̂* and Σ̂. However, the true parameters for the individual user would be *μ* and Σ and there exists a nearby untrained class with parameters *μ_other_* and Σ*_other_*. The numerical values for the distribution parameters are given in Table 3 and the data is illustrated in Figure 3. Using Equation (9) the probability density function for the yes-likelihoods (*p***_L_**_|_*_yes_*),

(15) $$\begin{array}{r} s_{1}={\left(z-\hat{\mu }\right)}^{T}{\hat{\Sigma }}^{-1}\left(z-\hat{\mu }\right)\\ z∼N(\mu ,\Sigma ) \end{array}$$

and the probability density function for the no-likelihoods (*p***_L_**|*_no_*),

(16) $$\begin{array}{r} s_{2}={\left(z-\hat{\mu }\right)}^{T}{\hat{\Sigma }}^{-1}\left(z-\hat{\mu }\right)\\ z∼N(\mu_{other},\Sigma_{other}) \end{array}$$

can be derived [24-27]. The CDF for the random variable *s_i_* is given by $P\{s_{i}\leq t\}=\sum_{k=0}^{\infty }c_{k}F(\cdot ,t)$ . The algorithm for the coefficients (*c*_0_ … *c_N_*) can be found from [19]. The likelihood distributions are shown in Figure 4 (top). The aim of the adaptation process (using Equation (10)) is to push the “no” distribution (*p***_L_**_|_*_no_*) to the right side of the *χ*_95_ threshold and the “yes” distribution (*p***_L_**_|_*_yes_*) to the left side. Assuming that the adaptation is perfect, the estimated parameters *μ̂*, Σ̂ equal the true parameters *μ*, Σ. Thus, the probability density function for the yes-likelihoods (*p***_L_**_|_*_yes_*) becomes

(17) $$\begin{array}{r} s_{3}={\left(z-\mu \right)}^{T}\Sigma^{-1}\left(z-\mu \right)\\ z∼N(\mu ,\Sigma ) \end{array}$$

and the probability density function for the no-likelihoods (*p***_L_**_|_*_no_*) become,

(18) $$\begin{array}{r} s_{4}={\left(z-\mu \right)}^{T}\Sigma^{-1}\left(z-\mu \right)\\ z∼N(\mu_{other},\Sigma_{other}) \end{array}$$

which are shown in Figure 4 (bottom). The chosen threshold is somewhat arbitrary, but it should be noted that in a real situation the parameters *μ_other_* and Σ*other* are not known, so it is difficult to avoid heuristics. However, the distribution for *s*_3_ Equation (18) is known in the case of perfect adaptation. This provides means to find a stopping criterion for the adaptation. For example, when a sample distribution function *S_n_*(*x*) of yes-likelihoods is sufficiently close to the optimal *χ*^2^ distribution function the adaptation process can be stopped. The measure for closeness can be, for example, Kolmogorov's *D_n_*,

(19) $$D_{n}=\underset{x}{\sup}|S_{n}(x)-F(x)|$$

where *F* is the known *χ*^2^ cumulative distribution function with *q* degrees of freedom.

### 6.3. A Confidence Measure

A fundamental requirement for sensor fusion is to obtain some kind of confidence measure for the decisions made. With this kind of information sensors can be be weighted based on the accuracy, or more sensors may be turned on if, for example, the uncertainty is high. We already described a likelihood based method that can be extended to the adaptation case easily: to obtain a confidence measure for the classifications after the adaptation, we will try to predict the probability of the user answering *yes*, given the current observed features and the adaptation data. To do this we use the Bayes' theorem,

(20) $$P_{yes|L}=\frac{p_{L|yes}P_{yes}}{p_{L}}$$

where *P_yes_*_|_**_L_** is the probability we are looking for, that is, the probability of the user answering *yes* given the likelihood value of the class that was selected. *p***_L_**_|_*_yes_* is the (empirical) conditional density of the yes-likelihood. *P_yes_* is the probability of successful detection after the adaptation. *p***_L_** is the weighted combination of the *yes* and *no* likelihoods. The *no* likelihood distribution enables us to normalize the posterior probability, which would be very difficult without the adaptation process [28]. It should be noted that in the case of the user answering *no*, we do not know the actual correct class. This is a drawback of the simple binary feedback method, but our method of predicting the probability of the *yes* answers does not require the knowledge of the true class in the case of a negative answer. To avoid heavy computations on the mobile phone, the required distribution estimates can be computed on the server side. The necessary information to be transmitted to the server would be the *L_i_*(*yes*) and *L_i_*(*no*) values, and the server would return the new classifier parameters along with *p***_L_**_|_*_yes_*,*P_yes_* and *p***_L_**.

## 7. Results and Discussion

We implemented and tested three different classifiers: an indoor/outdoor classifier, an environment classifier, and an activity classifier. In addition to the maximum a posteriori (MAP) classification method presented in Section 5 we present results using decision trees (DT) and support vector machines (SVM) for comparison. For those purposes MatLab's *ClassificationTree class* with default options and SVM functions with linear kernel function were used. In the case of SVM, one-versus-others classifiers were implemented and the class was selected based on the classifier which classified the test data with the greatest margin. Evaluation of the accuracy of the all algorithms was first done by using leave-one-user-out cross validation, where at each round all the data from a user is held out and the system is trained with the data from the remaining users. This way we can ensure that the system is not overfitting to the individual characteristics of any user. As the training data was collected in opportunistic way, the class imbalance problem [29] may occur. Thus, equal priors were given for all classes in MAP classifier as we cannot prefer any class over another.

In the second phase, to evaluate our adaptation method presented in Section 6 the second data collection campaign was arranged and the method for classifier adaptation was evaluated.

### 7.1. Indoor/outdoor Classification

A study to evaluate the accuracy of a simple {*indoor, outdoor*} classifier was performed. In this task GPS signals are very relevant as the low power levels prevent satellite signal tracking in most buildings. The binary classification induces some problems in defining the boundaries. We defined driving a passenger car or travelling in a train as *outdoor* environment, the main reason being that radio receivers are less affected by vehicles than buildings.

The confusion matrix for the MAP classifier is presented in Table 4.

Especially good features for differentiating between these classes were the WLAN signal strengths, which are usually higher indoors than outdoors. By using only the WLAN signal strength we could obtain classification accuracies of 78% and 82% for indoor and outdoor, respectively. Nevertheless, the results may become very different if the data would be collected only from areas where the WLAN station density is low.

By using DT and SVM we could achieve higher classification rates for the *indoor* and *outdoor* class. Confusion matrices, where all the features from GPS, WLAN and Bluetooth were used, are presented in Tables 5 and 6 for DT and SVM, respectively. This shows that the decision tree gives the highest classification rate with these classes. However, to test our adaptation method we continued to use MAP algorithm also for these classes.

### 7.2. Environment Classifier

For environment classification we selected the set of {*restaurant/pub/cafe, office, home, street/road, nature*} as the categories to be recognized. The list includes environments that would be useful for many mobile applications, but it is short enough for reasonable accuracy analysis. In environment classification all the features from the GPS, WLAN and Bluetooth were selected. In addition, the likelihoods output by the audio classifier were used as features. Features from the accelerometer were not used in this case as they did not improve the recognition accuracy.

**Figure 5.**
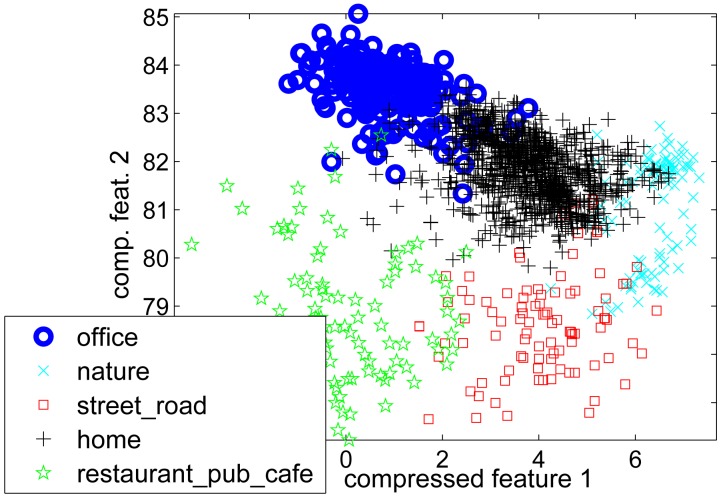
Two main features after the feature compression.

Figure 5 shows the scatter plot of the first two features after the compression method presented in Section 4 was done. Some of the most important features were audio features and GPS TTFF and maximum WLAN signal strength. The confusion matrix of leave-one-user-out cross-validation method is presented in Table 7.

DT and SVM results for the classes *office*, *nature*, *street/road*, *home* and *restaurant/pub/cafe* are presented in Table 8. As the some classes

Class average classification accuracy for MAP classifier is 70%, for DT 68% and for SVM 63%. Thus, there are no great differences between these classifiers. It should be noted that default parameters from MatLab's *ClassificationTree class* were used for DT and SVM, in addition, the class imbalance problem was not taken into account for these classifiers, thus by taking these aspects into account, accuracies may slightly increase. However, the main idea in the paper was not to show whether the Bayesian classifier can beat DT or SVM, but to show that our Bayesian classifier is not dramatically inferior to state of art methods and thus it is feasible to propose adaptation algorithm for this Bayesian approach. Comprehensive comparative between classifiers is thus left out.

### 7.3. Activity Classifier

The following classes were included in the activity recognition:

- table; phone is placed on a non-moving surface
- standing; phone is in the hand or pocket, but the user is not walking or running
- walking; the user is walking
- running; the user is running
- bicycling; the user is bicycling
- vehicles; the user is driving or traveling with a motorized vehicle

It is quite clear that the GPS speed would be a very important feature for this kind of classification. However, reliable speed information is not available all the time, so the activity classifier was implemented with two different modes, depending on the availability of the GPS [30].

In this kind of activity classifier, the classes *table*, *standing*, and *vehicles* may overlap in some cases. Reason for this is that the variance of the accelerometer data norm, which is usually the most dominant feature in the activity classifier, can be similar in some cases. For example, a car stopped in traffic lights or a person standing still can be confused as the class on *table*. Nevertheless, if we also use features from the GPS (GPS speed) and causal information based on, *e.g.*, markov chain modelling of the classification outputs ([31]), the separation of a car stopped in traffic lights and standing can usually be done.

The original data set did not contain enough data for the results with the activity classifier when the GPS speed is available. Thus, data were generated where the standard deviations (std) and means of the GPS speeds for each activity were chosen manually. This made it impossible to perform the decision tree classification but enabled us to calculate the adaptation results also for the activity classifier. The values selected are shown in Table 10. It should be noted that the values are naturally changing depending on the user. However, our adaptation algorithm can scale these values for the individual use.

### 7.4. Adaptation Results

**Figure 6.**
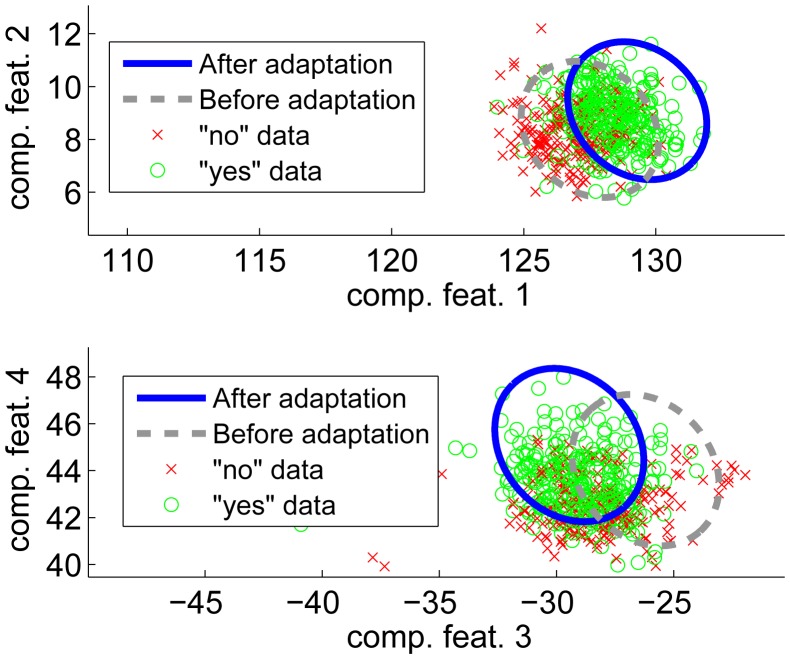
Class *street/road* features.

**Figure 7.**
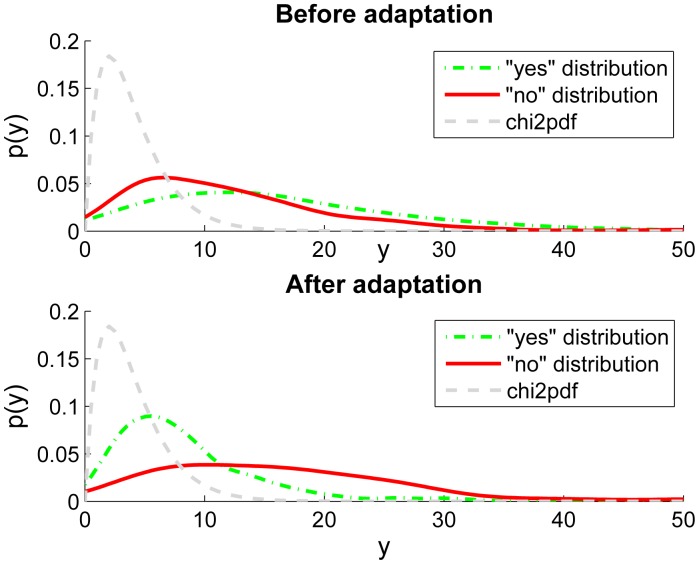
Class *street/road* probability distribution functions.

For the test persons, carrying the phones and answering yes or no to the recognition results does not require a big effort, but the results show that the information obtained is very valuable in both adaptation and evaluation of the classifier performance. As an example, a scatter plot of the two most important transformed features of the data from the class *street/road* is shown in Figure 6. In the upper panel, the original 95 percent confidence ellipse (The ellipse containing 95% of the mass of the Gaussian probability density function when using the estimated covariance calculated from the training data) of the *street/road* training data is depicted using a dashed line, and the ellipse after the adaptation in solid line. The data with *yes*-tag is plotted with small circles, and the data with *no*-tag with small crosses. The bottom panel of Figure 6 is the same for the features three and four. Figure 7 shows the estimated probability density functions (*p*(*y*)) obtained using Matlab's *ksdensity* function. The figure shows the *yes*-tag likelihoods (dash-dotted) and *no*-tag likelihoods (solid line) before and after the adaptation. The dashed curve is the Chi squared probability density function which would be the optimal distribution for the *yes*-tag likelihoods. This means that if our model would be correct and our assumption of the Gaussian distributed features would be true, the dash-dotted curve (“yes” distribution) should follow the dashed curve (chi2pdf). One can clearly see that there is not much difference between the *yes*- and *no*-likelihoods before adaptation (upper plot in Figure 7). This means that we cannot assign a confidence measure based on these likelihoods alone. The reason is that the original training data was not sufficient to obtain accurate class parameters or the adaptation data is collected in an environment having different features compared to the training data. After the adaptation is completed, the bottom plot in Figure 7 shows a clear improvement: the *no*-likelihoods are visibly separated from the *yes*-likelihoods.

After the adaptation round is complete, a convenient way to estimate the accuracy of the classifiers is to use the prior probabilities, *P*(*user answers yes*), using Equation (20). To evaluate accuracy of the proposed adaptation method the data from the very first data collection campaign was used (Section 2.1). Ten users with the most data were selected and the classifier was trained leaving one user out of the training data set at the time. Data from this user was then divided to half, other half containing test set for classifier and other half was used to create virtual *yes* and *no* answers using. This enabled us to know also the ground truth of the *no* answers (which was not available in the second data collection campaign presented in Section 2.2). Table 11 presents the classification data of these ten users before the adaptation was applied. Classifier precision and recall are 50% and 67% , respectively. The results after the adaption are presented in Table 12. In this case precision is 68% and recall 70%. Naturally, the *no* answers were more valuable information for the adaptation process. It was noticed that already couple of *no* answers could improve the class parameters.

### 7.5. Confidence Measure Results

**Figure 8.**
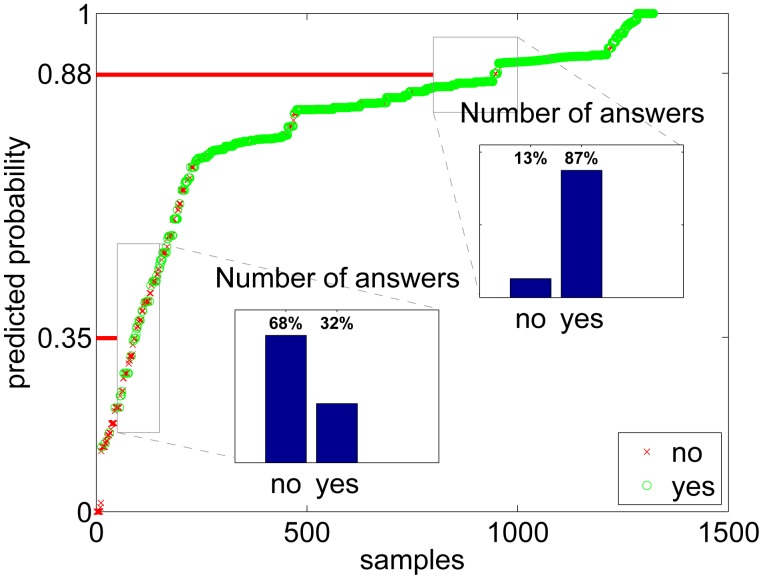
Predicted *yes*-probability for each sample in the sorted activities data. The small bar plots tell how many yes and no answers there are inside the two small rectangural areas.

**Figure 9.**
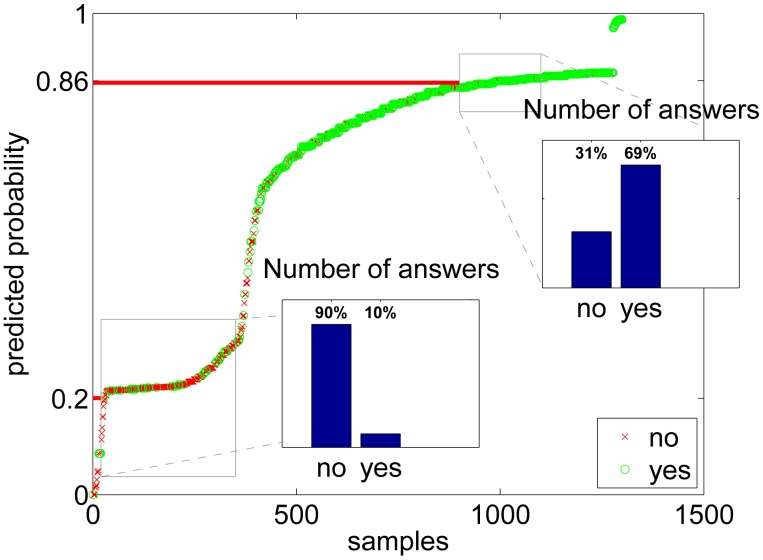
Predicted *yes*-probability for each sample in the sorted environment data. The small bar plots tell how many yes and no answers there are inside the two small rectangural areas.

To show that the estimated probabilities of *yes*-answers actually resemble the true probabilities, a rough visualization is made for the activities in Figure 8 and for the environments in Figure 9. In the figures, all the answers from all users are sorted by the estimated probability, and the results are plotted using a circle if the user answer was *yes* and with a cross in the case of a *no* answer. To see if the prediction of probability is approximately working, we can then zoom into some probability level and see if the distribution between the circle (*yes*)) values agrees with the y-axis number. The lower box of Figure 8 shows an example where the predicted probability level of our model is 0.35 and the actual amount of yes answers in the data is 32%. Respectively, the higher box depicts a situation where our model predicts a proportion of yes answers to be 0.88, whereas the actual proportion of yes labeled samples in the data is 87%. This approximation shows that the estimation is working very well for activities, however, for environments (Figure 9) there are more differences in predicted probability compared to the estimate calculated using yes and no answers.

**Figure 10.**
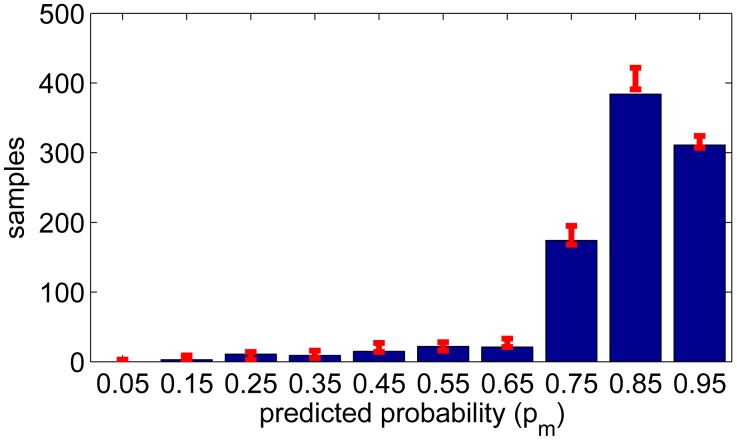
The number of samples (yes answers) at each bin centered at *p_m_* and 95% limits from Binomial distribution (activities).

**Figure 11.**
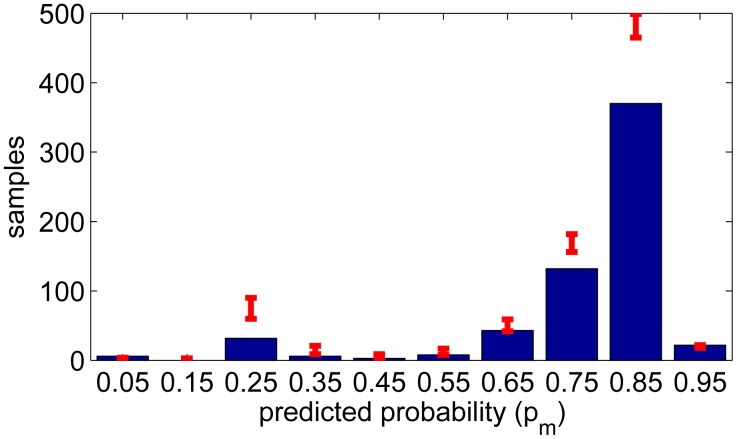
The number of samples (yes answers) at each bin centered at *p_m_* and 95% limits from Binomial distribution (environments).

To analyze the result more in detail, we also show the limits *t_l_,t_u_* around the mean

(21) $$P(t_{l}\leq \theta <t_{u})=0.95,$$

with *θ* ∼ *Bin*(*N*(*yes*) + *N*(*no*)*,p_m_*) for each bin to see how the samples would deviate with perfectly known *p_m_*. The limits are shown in Figure 10 for the activities and Figure 11 for the environments, where the data is divided into 10 boxes having the middle points *p_m_* = 0.05 . . . 0.95. The height of each bin is the number of yes answers satisfying |*p_m_* − *P_yes_*_|_**_L_**| < 0.05. This result shows that for the activities the estimated confidence measure is consistent. However, for the environments the confidence measure is slightly optimistic.

## 8. Conclusions

We have presented an activity and environment recognition implementation suitable for modern mobile phones. Two data collection campaigns were organized, the first for the background training data and the second for testing the adaptation algorithms. The initial design goal of having an adaptive system that can provide confidence measures along with the classification results was met by developing a Bayesian approach that utilizes binary user feedback.

The results show that some important environments and activities can be recognized with reasonable accuracy, but individual adaptation is very likely needed for applications requiring context information. In addition, the adaptation is needed for providing a proper confidence measure for the classification result. Furthermore, the paper showed that the confidence measure is consistent with the selected set of activities classes. For the selected environments, the confidence measure was slightly optimistic.

Our implementation enables an individual adaptation in a mobile device. To avoid heavy computations on the mobile device, the required distribution estimates can be computed on the server side. The only necessary information to be transmitted to the server would be the set of likelihood values corresponding to observations with a yes-label and a no-label.

## Figures and Tables

**Table 1.** Data trial statistics.

**Total Number of Users**	21
Number of men	15
Number of women	6
Median age	31
Mean age	32.3
Total number of annotations	3478
Number of unique annotations	620

**Table 2.** Collected data.

**Sensor**	**Sampling Rate**	**Sample Length**
3-axis Accelerometer	100 Hz	1 min
GPS	1 Hz	2 min
WLAN	$\frac{1}{12}\text{Hz}$	1 min
Bluetooth	recorded once	snapshot
GSM/3G Cell information	1 Hz	1 min
Audio	16 kHz	1 min

**Table 3.** Parameters used in the simulation.

**Parameter**	**Value**
*μ*	[4 6]
Σ	$$\left[\begin{array}{cc} 0.8 & 0.5\\ 0.5 & 0.7 \end{array}\right]$$
*μ̂*	[1 2]
Σ̂	$$\left[\begin{array}{cc} 0.4 & -0.2\\ -0.2 & 1.2 \end{array}\right]$$
*μ_other_*	[0 0]
Σ*_other_*	$$\left[\begin{array}{cc} 2 & -0.6\\ -0.6 & 1 \end{array}\right]$$

**Table 4.** Confusion matrix of the classes *indoor* and *outdoor* using MAP classifier. The values denote percentages.

**true**	**In**	**out**

**decision**		
**In**	82	18
**Out**	18	82

**Table 5.** Confusion matrix of the classes *indoor* and *outdoor* using decision trees. The values denote percentages.

**true**	**In**	**out**

**decision**		
**In**	96	4
**Out**	6	94

**Table 6.** Confusion matrix of the classes *indoor* and *outdoor* using SVM. The values denote percentages.

**true**	**In**	**out**

**decision**		
**In**	86	14
**Out**	6	94

**Table 7.** Confusion matrix of five environment classes *office*, *nature*, *street/road*, *home* and *restaurant/pub/cafe* using MAP classifier. The values denote percentages.

**true**	**Off**.	**Nat**.	**Str**.	**Home**	**Res**.

**decision**					
**Off**.	75	0	2	7	17
**Nat**.	0	67	21	5	8
**Str**.	1	9	72	6	12
**Home**	3	5	15	62	15
**Res**.	7	0	21	0	73

**Table 8.** Confusion matrix of five environment classes *office*, *nature*, *street/road*, *home* and *restaurant/pub/cafe* using the decision tree classifier. The values denote percentages.

**true**	**Off**.	**Nat**.	**Str**.	**Home**	**Res**.

**decision**					
**Off**.	70	0	1	20	10
**Nat**.	2	65	20	6	7
**Str**.	2	12	62	11	13
**Home**	4	2	9	77	8
**Res**.	2	1	26	4	68

**Table 9.** Confusion matrix of five environment classes *office*, *nature*, *street/road*, *home* and *restaurant/pub/cafe* using the support vector machine classifier. The values denote percentages.

**true**	**Off**.	**Nat**.	**Str**.	**Home**	**Res**.

**decision**					
**Off**.	87	0	0	5	8
**Nat**.	2	36	26	23	13
**Str**	3	13	56	8	20
**Home**	2	5	5	73	16
**Res**.	18	3	8	7	65

**Table 10.** The means and standard deviations of the GPS speeds that were used in the activity classifier.

	**run**.	**bicyc**.	**walk**.	**stand**.	**table**	**vehic**.
**Mean(km*/*h)**	7	15	4	0.5	0.5	60
**Std(km*/*h)**	2	5	2	0.5	0.5	30

**Table 11.** Confusion matrix of five environment classes *office*, *nature*, *street/road*, *home* and *restaurant/pub/cafe* before adaptation.

**true**	**Off**.	**Nat**.	**Str**.	**Home**	**Res**.

**decision**					
**Off**.	76	0	2	11	11
**Nat**.	0	55	35	5	5
**Str**.	3	8	78	0	11
**Home**	3	1	17	65	14
**Res**.	11	0	30	0	59

**Table 12.** Confusion matrix of five environment classes *office*, *nature*, *street/road*, *home* and *restaurant/pub/cafe* after adaptation.

**true**	**Off**.	**Nat**.	**Str**.	**Home**	**Res**.

**decision**					
**Off**.	81	0	1	6	13
**Nat**.	0	60	20	5	15
**Str**.	3	5	73	5	14
**Home**	3	1	9	74	13
**Res**.	11	0	25	0	64
